# Two-legged hopping in autism spectrum disorders

**DOI:** 10.3389/fnint.2013.00014

**Published:** 2013-03-26

**Authors:** Matthew F. Moran, John T. Foley, Mary E. Parker, Michael J. Weiss

**Affiliations:** ^1^Department of Physical Therapy and Human Movement Science, Sacred Heart UniversityFairfield, CT, USA; ^2^Department of Physical Education, State University of New York at CortlandCortland, NY, USA; ^3^Department of Physical Therapy, Texas State UniversitySan Marcos, TX, USA; ^4^Department of Psychology, Fairfield UniversityFairfield, CT, USA

**Keywords:** sensory processing, autism spectrum disorder, motor control, proprioception, stiffness

## Abstract

Sensory processing deficits are common within autism spectrum disorders (ASD). Deficits have a heterogeneous dispersion across the spectrum and multimodal processing tasks are thought to magnify integration difficulties. Two-legged hopping in place in sync with an auditory cue (2.3, 3.0 Hz) was studied in a group of six individuals with expressive language impaired ASD (ELI-ASD) and an age-matched control group. Vertical ground reaction force data were collected and discrete Fourier transforms were utilized to determine dominant hopping cadence. Effective leg stiffness was computed through a mass-spring model representation. The ELI-ASD group were unsuccessful in matching their hopping cadence (2.21 ± 0.30 hops·s^−1^, 2.35 ± 0.41 hops·s^−1^) to either auditory cue with greater deviations at the 3.0 Hz cue. In contrast, the control group was able to match hopping cadence (2.35 ± 0.06 hops·s^−1^, 3.02 ± 0.10 hops·s^−1^) to either cue via an adjustment of effective leg stiffness. The ELI-ASD group demonstrated a varied response with an interquartile range (IQR) in excess of 0.5 hops·s^−1^ as compared to the control group with an IQR < 0.03 hops·s^−1^. Several sensorimotor mechanisms could explain the inability of participants with ELI-ASD to modulate motor output to match an external auditory cue. These results suggest that a multimodal gross motor task can (1) discriminate performance among a group of individuals with severe autism, and (2) could be a useful quantitative tool for evaluating motor performance in individuals with ASD individuals.

## Introduction

Individuals diagnosed with autism spectrum disorders (ASD) not only demonstrate language, social and sensory impairments but also movement abnormalities (DSM-IV, 2000). In fact, movement abnormalities may be the hallmark of many diagnoses as restricted, repetitive, and stereotypical movements are commonly observed in individuals with ASD. Motor impairments of children/adults with autism may include gross motor coordination (e.g., Calhoun et al., 2011), fine motor coordination (e.g., Gernsbacher et al., 2008), motor stereotypies (e.g., Loh et al., 2007), postural control (e.g., Molloy et al., 2003; Minshew et al., 2004), and/or motor apraxia (e.g., Ming et al., 2007). A recent meta-analysis concluded that motor impairments are present across the spectrum with deficiencies reported in motor planning, sensorimotor integration, and motor execution (Fournier et al., 2010). Inquiry into these movement aberrations appears warranted as these motor impairments may exceed other ability areas and influence both language and social integration (Piek and Dyck, 2004).

Sensory processing deficiencies are commonly associated with ASD (Tomchek and Dunn, 2007) with prevalence estimates ranging from 30 to 100% of respective study participants (Dawson and Watling, 2000). Following a meta-analysis of 14 relevant studies, Ben-Sasson et al. (2008) concluded that “under-responsivity,” delayed or muted response to a stimuli, was reported more by parents of children with ASD than either “over-responsivity” or “seeking” out of stimuli. Several recent reports point to the processing deficiencies of visual, auditory, tactile and proprioceptive stimuli in individuals with autism (Jasmin et al., 2008; Orekhova et al., 2012; Paton et al., 2012). These hypo-responses may actually be the result of increased sensitivity to stimuli rather than the opposite (Rinaldi et al., 2008). Through various work on a valproic acid rat model of autism, Markram et al. (2007) suggests that both increased response to stimuli and increased plasticity of neuronal circuits may explain altered responses observed in ASD. While it could be argued whether these sensory processing deficits are a core feature of ASD or a co-morbidity, it is apparent that they are present in a large percentage of individuals with ASD and they impact communication, social interaction, and movement qualities.

Propioceptive deficits in individuals with ASD have received less inquiry than other sensory types, although proper joint and limb positioning is critical for movement precision. Afferent proprioceptive feedback is primarily afforded from golgi tendon organs, muscle spindles, joint receptors, and skin receptors. This feedback is critical during all forms of human location (e.g., running, walking, hopping) as the leg acts as a tuned spring that can store and return a certain percentage of energy (Farley et al., 1991; Ferris and Farley, 1997). During landing the leg spring is compressed storing energy and during propulsion the leg spring rebounds as the joints (hip, knee, ankle) extend. Leg spring stiffness is actively controlled as both a factor of locomotion speed and ground surface compliance in order to minimize overall energetic cost. Propioceptive feedback is necessary to essentially “tune” leg spring stiffness and maximize the amount of returned energy. When children with autism learn a novel task, there is a stronger association between proprioceptive feedback and self-generated motor commands than seen in typically developing children (Haswell et al., 2009). Haswell et al. (2009) speculate that overexpression of cortical connections between the somatosensory cortex and primary motor cortex may explain the increased reliance on proprioceptive feedback in their generalized motor internal model. Altered proprioceptive feedback has also been cited as a potential cause of motor dyspraxia observed in individuals with Asperger syndrome (Weimer et al., 2001).

In contrast to these findings in Asperger syndrome, Fuentes et al. (2011) recently showed children with ASD displayed motor impairment without any deficits in proprioception during a simple upper extremity elbow flexion-extension task. These are compelling results because they may indicate that proprioceptor sensors are neither hyper- or hypo-sensitive in individuals with ASD and it is the rather the integration of proprioceptive information with other sensory inputs (e.g., visual, auditory, vestibular-proprioceptive information) that may be impaired. High functioning individuals with autism have previously demonstrated a delayed motor anticipation response and an inability to decrease reaction time when presented with a visual cue during a button pressing task (Rinehart et al., 2001). This increased temporal processing seems to be exacerbated in individuals with ASD during conditions of multisensory input (Kwakye et al., 2011).

Synchronizing motor output with an auditory cue, sensorimotor synchronization, has been studied extensively via a finger-tapping model (e.g., Kelso, 1984; Ivry and Keele, 1989; Sheridan and McAuley, 1997) but whole body rhythmicity has received much less attention (Rousanoglou and Boudolos, 2006). Timing of rhythmic movement has been explained via a (1) two-stage timing model (Wing and Kristofferson, 1973) and a (2) dynamic system model (Schöner, 2002). Utilizing the two-stage model of synchronization, Ivry and Keele (1989) discovered that individuals with cerebellar lesions had disruptions of their internal clock variance but not motor error variance during an auditory-cued finger tapping task. Similarly, Sheridan and McAuley (1997) reported that ASD children were less accurate and more variable with finger tapping precision than control groups. Although the two-stage timing model has been used to explain timing and motor errors during finger tapping, Rousanoglou and Boudolos (2006) found that timing control during an auditory-cued two-legged hopping in place task could be explained via a dynamic systems model. The authors speculate that alteration of joint stiffness may modify the rate of ground reaction force development (RFD) during the landing phase and that RFD may serve as a timing regulator. No previous work has examined whole body sensorimotor synchronization in ASD.

It is also noteworthy that the most extreme differences or disorders of movement regulation and/or regulation of proprioceptive feedback may correlate with the “severity” of ASD. Donnellan et al. (2010) present evidence that disorders of sensory processes and movement are endemic to all forms of ASD. However, the evidence that they present raises the inquiry of whether individuals who have the most compromised forms of “self advocacy” such as significant expressive language challenges also present with more profound differences in a range of sensory-movement anomalies (Hill and Leary, 1993; Donnellan et al., 2006, 2010). Furthermore, there remains the need to differentiate the developmental presentations across the range of individuals who have differing forms of an ASD diagnosis.

Therefore, the purpose of this study was to investigate whether individuals with ASD with expressive language impairments (ELI-ASD) could modify their motor control strategy during a multi-joint gross motor activity (two-legged hopping in place) to match an auditory cue (temporal synchrony). It was hypothesized that:

H(1) The individuals with ELI-ASD would be able to successfully complete a two-legged hopping in place task at a self-selected cadence.

H(2) The individuals with ELI-ASD population would not match their hopping cadence to an external auditory cue while all control participants would be within 5% of the cue.

H(3) There would be a range of responses within the ELI-ASD population.

The results of this study may potentially further our understanding of sensory processing deficits in this population and provide a basis for a quantitative movement assessment screening tool that could be used to evaluate intervention efficacies and better classify individuals with ASD.

## Materials and methods

### Participants

Nine individuals diagnosed with expressive language impairments autism spectrum disorders (ELI-ASD) were recruited for this study. A case-control study design was used because of the small sample size due to difficulties recruiting and testing in the ELI-ASD population. Ten age-matched control participants were recruited for this study (Table 1). An independent *t*-test was conducted to confirm that the groups were appropriately matched for age (*p* > 0.05). All participants were screened for musculoskeletal injury that would influence their ability to complete the study's protocol. The experimental protocol was approved by the Institutional Review Board at Sacred Heart University and informed consent was obtained from all participants/guardians prior to data collection. Inclusion criteria for the ELI-ASD group was determined by a Childhood Autism Rating Scale (CARS) score greater than 37 indicating “severely autistic” and at least a rating of 3 out of 4 on Sub-Scale XI “Verbal Communication,” which indicates a severe disorder of verbal behavior such as not speaking in more than a few words or phrases; routinely not using verbally produced sentences (Schopler et al., 1980). All of the experimental participants met the “severely autistic” criteria, with the group showing a mean total score of 50.1 ± 5.6, and all presented with severe disorders of verbal communication (indicated by a rating of “4” out of 4 for all participants). All participants were evaluated by the same investigator (MJW), who is trained in CARS implementation and has over 30 years of experience, to determine inclusion within this study. Three participants in the initial ELI-ASD group did not complete the study due to behavioral and/or attention issues.

**Table 1:** **Subject demographics**.

	**Control**	**ELI-ASD**
Age (years)	19.7 ± 0.5	18.8 ± 2.1
Gender	7M, 3F	6M, 0F
Mass (kg)	78.3 ± 8.3	83.9 ± 15.4
CARS	na	50.1 ± 5.6

The groups were not significantly different for either age or weight (p > 0.05). The Childhood Autism Rating Scale (CARS) was used to assess level of autism in ELI-ASD subjects. [Mean ± SD].

### Experimental protocol

Participants were first positioned on a ground mounted force plate (40.6 × 81.2 cm) (Model OR6-5, Advanced Medical Technology, Inc.; Watertown, MA) with feet shoulder-width apart and hands placed on their hips. Each hopping trial lasted 15 s and participants hopped in place for the entire trial. Pilot testing determined that 15 s was to be an adequate length of time to allow participants enough hops to become in sync with auditory cue but not too long where fatigue would set in. Any trial where the participant did not land on the force plate was discarded and re-collected. Vertical ground reaction force (*v*GRF) was collected from the force place at a sampling frequency of 200 Hz. For the first two trials, participants were asked to perform two-legged hopping at a self-selected frequency. No instructions were given as to how high to hop. Before the first trial a researcher stood approximately 1-meter anterior to each participant and demonstrated two-legged hopping in place.

For the remaining four trials, participants were given 10 s to listen to a metronome prior to stepping on the force plate and attempting to hop in unison with the auditory cue. Although some individuals with autism may demonstrate a hyper-auditory response, a metronome was selected for the current study as its use has previously occurred as an interactive intervention with this population (Mays et al., 2011; Kim et al., 2012). Auditory cues were randomized and were set at either 2.3 or 3.0 Hz, as these frequencies typically do not correspond to previously reported normative two-legged hopping frequencies (Farley et al., 1991; Rousanoglou and Boudolos, 2006). The research design was intended to force participants to hop at non-preferred cadences. In between hopping trials, each subject was given 2 min to rest. During *post-hoc* analysis, a trial was scored as “successful” if the actual hopping cadence deviated from the auditory cue frequency by less than 5% (Granata et al., 2002).

### Data analysis

**Figure 1:**
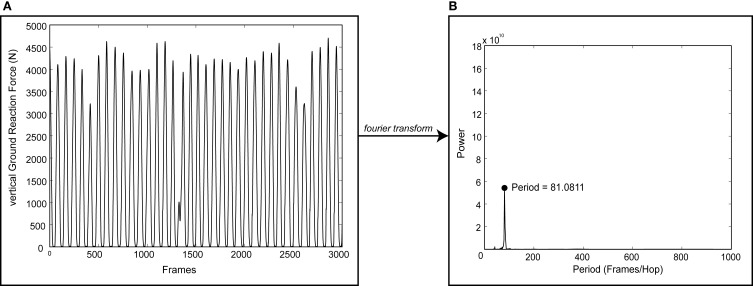
**Sample output of the process used to determine dominant hopping cadence.** A discrete Fourier transform was applied to vertical ground reaction force data (*v*GRF, **A**) to determine the dominant hopping cadence **(B)** for each trial. Note the discontinuities in *v*GRF indicating an inconsistent hopping pattern.

Data was exported to MATLAB software (MathWorks, Inc; Natick, MA) for post-processing. *v*GRF data were digitally filtered using a 4th order Butterworth low-pass filter with cutoff frequency of 50 Hz. A discrete Fourier transform was applied to the vGRF data to convert it into the frequency domain. The dominant frequency (i.e., hopping cadence) for each trial was then determined (Figure 1). This frequency analysis was preferred because it computed the dominant cadence over the 15 s data collection window regardless if there were inconsistencies in the hopping motion. Deviation (*d*) percentages were computed for each trial as the absolute difference between cued frequency (ω_cue_) and actual hopping frequency (ω_actual_) divided by the cued frequency.

(1) $$d=\left(|\omega_{\text{cue}}-\omega_{\text{actual}}|/\omega_{\text{cue}}\right)\times 100$$

**Figure 2:**
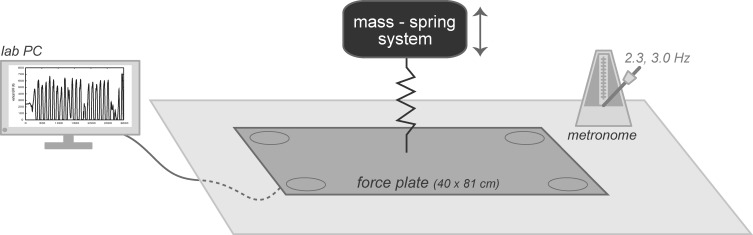
**Subjects were positioned on a 40 × 81 cm embedded force plate, a metronome was set at either 2.3 or 3.0 Hz, and a lab computer collected vertical ground reaction forces.** The system was modeled as a mass-spring system where effective leg stiffness was calculated for each trial.

Two-legged hopping in place at a frequency ≥ 2.2 hops·s^−1^ has previously been demonstrated to behave as a simple mass-spring system (Figure 2) (Farley et al., 1991). Effective leg stiffness (*k*), representative of the musculotendon stiffness, was subsequently calculated from both the time duration and *v*GRF during landing and takeoff (Farley et al., 1991).

(2) k=vGRF×(2π/T)

### Statistical analysis

Differences in hopping cadence and effective leg stiffness were assessed for significance utilizing two mixed factorial ANOVA's (group by auditory cue, group by effective leg stiffness) with group membership as the between-subject factor and hopping cadence and effective leg stiffness as the within-subject factors. *Post-hoc* analysis to determine differences in hopping cadence or effective leg stiffness between groups was conducted using independent *t*-test. Paired *t*–test were used to identify within group differences in hopping cadence and effective leg stiffness. Statistical significance was set apriori with a significance level of α = 0.05. To correct for multiple *t*-test, a Holm's Sequential adjustment was employed (Holm, 1979). All statistical analysis was computed in PASW Statistics 18 (Chicago, IL). Interquartile ranges (IQR) were computed for hopping cadencies as IQR is measure of central tendency that is resistant to outliers.

## Results

Mauchly's Test for Sphericity indicated that the assumption of sphericity had been violated for the main effect of auditory cue on hopping cadence, χ^2^_(2)_ = 16.75, *p* < 0.001, therefore, degrees of freedom were corrected using a Greenhouse-Geisser estimate (Field, 2009). There was a significant main effect of auditory cue on both hopping cadence *F*_(1.16, 16.24)_ = 12.26, *p* < 0.05 and effective leg stiffness *F*_(2, 28)_ = 11.67, *p* < 0.001. Additionally, there was a significant interaction effect between group membership and auditory cue on hopping cadence *F*_(1.16, 16.24)_ = 4.97, *p* < 0.05 and between group membership and auditory cue on effective leg stiffness *F*_(2, 28)_ = 3.60, *p* < 0.05. This interaction highlights the importance of investigating the two different groups.

### Self-selected cadence

**Figure 3:**
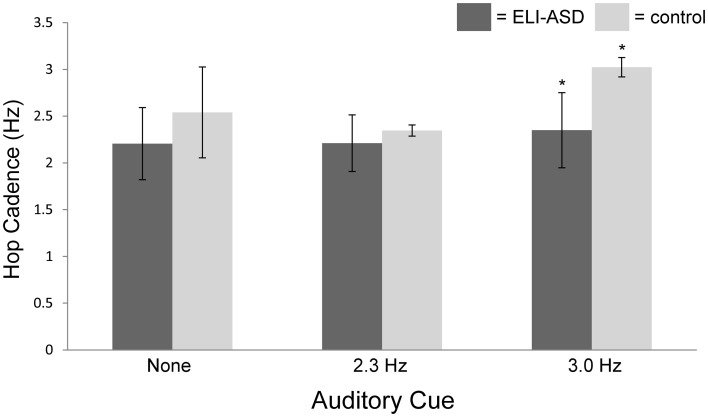
**Hop cadence vs. auditory cue (none, 2.3 Hz, 3.0 Hz).** The control group was successful in 95% of trials in matching auditory cue while the ELI-ASD group was successful in only 33% of cued trials. The control group was significantly better at matching the 3.0 Hz auditory cue than the ELI-ASD group (*p* < 0.05).

At their self-selected cadence, participants in the control group hopped at 2.54 ± 0.49 hops·s^−1^ (IQR = 0.37 hops·s^−1^) (Figure 3) with an effective leg stiffness of 29.8 ± 6.5 kN·m^−1^ (Table 2). Participants with ELI-ASD hopped at a cadence of 2.21 ± 0.44 hops·s^−1^ (IQR = 0.59 hops·s^−1^) with an effective leg stiffness of 29.2 ± 8.6 kN·m^−1^. *Post-hoc* analysis revealed there were no statistically significant differences between groups in their hop cadence, *t*_(14)_ = 1.41, *p* > 0.05, *r* = 0.35; and in effective leg stiffness, *t*_(14)_ = 0.38, *p* > 0.05, *r* = 0.10.

**Table 2:** **Effective leg stiffness (kN·m^−1^) for Control and ELI-ASD groups. [Mean ± SD]**.

	**Auditory cue**		
	**Self-selected**	**2.3 Hz**	**3.0 Hz**
Control (*n* = 10)	29.8 ± 7.0	28.2 ± 7.3	40.6 ± 6.9
ELI-ASD (*n* = 6)	29.2 ± 8.6	30.8 ± 6.2	33.9 ± 6.7

### Auditory cue 1—2.3 Hz

On average, the control group only deviated from the 2.3 Hz cue by 2.6% (2.35 ± 0.06 hops·s^−1^) with 95% of all collected trials successful (<5% deviation). In contrast, the ELI-ASD group deviated, on average, by 7.6% (2.21 ± 0.30 hops·s^−1^) for the 2.3 Hz cue trials and only 42% of all collected trials were deemed successful. IQR for the control group during the 2.3 Hz trials was 0.06 hops·s^−1^ as compared to 0.43 hops·s^−1^ for the ELI-ASD group. Effective leg stiffness values were 28.2 ± 7.3 kN·m^−1^ and 30.8 ± 6.2 kN·m^−1^, respectively, for the control and ELI-ASD groups. A Levene's Test for Equality of Variances indicated that assumption was not met for hopping at 2.3 Hz, *F*_(1, 14)_ = 15.29, *p* < 0.001. *Post-hoc* analysis revealed there were no statistically significant differences between groups for hop cadence, *t*_(5.19)_ = 1.15, *p* > 0.05, *r* = 0.45; and in effective leg stiffness, *t*_(14)_ = −0.63, *p* > 0.05, *r* = 0.17.

### Auditory cue 2—3.0 Hz

The control group had similar performance during the 3.0 Hz cueing trials with an average deviation of 2.5% and a 95% success rate. In contrast the ELI-ASD group deviated by 21.7% and had a 25% success rate. Effective leg stiffness increased to 40.6 ± 6.9 kN·m^−1^ in the control group and 33.9 ± 6.7 kN·m^−1^ in the ELI-ASD group. The control group IQR for hopping cadence was 0.00 hops·s^−1^ compared to 0.71 hops·s^−1^ in the ELI-ASD group. *Post-hoc* analysis revealed that participants in the control group hopped at a significantly higher cadence *t*_(14)_ = 5.68, *p* < 0.001, *r* = 0.84; and with more effective leg stiffness, *t*_(14)_ = 2.15, *p* = 0.05, *r* = 0.50.

### Within group comparisons

When comparing within each group between their self-selected cadence and 2.3 or 3.0 Hz auditory cue conditions, the control group exhibited a significantly different hop cadence between 3.0 Hz and the self-selected cadence, *t*_(9)_ = 3.43, *p* < 0.01, *r* = 0.75. This difference was also seen in effective leg stiffness between 3.0 Hz and the self-selected cadence *t*_(9)_ = −4.69, *p* = 0.001, *r* = 0.84. However, the control group had no statistical difference between self-selected cadence and the 2.3 Hz in both hopping, *t*_(9)_ = 1.31, *p* > 0.05, *r* = 40; and effective leg stiffness, *t*_(9)_ = 0.62, *p* > 0.05, *r* = 0.20. A comparison between the 2.3 and 3.0 Hz auditory cues in the control group revealed a significant difference existed between the two cues both in hopping, *t*_(9)_ = −29, *p* < 0.001, *r* = 0.99; and effective leg stiffness, *t*_(9)_ = −4.84, *p* = 0.001, *r* = 0.85.

Participants in the ELI-ASD group did not significantly alter either hop cadence between both the self-selected cadence and the 2.3 Hz condition, *t*_(5)_ = −0.09, *p* > 0.05, *r* = 0.04; and the self-selected cadence and the 3.0 Hz condition, *t*_(5)_ = 1.05, *p* > 0.05, *r* = 0.43. This pattern was also apparent in leg stiffness where there was no significant difference found between both the self-selected cadence and the 2.3 Hz condition, *t*_(5)_ = −1.16, *p* > 0.05, *r* = 0.46; and the self-selected cadence and the 3.0 Hz condition, *t*_(5)_ = −1.55, *p* > 0.05, *r* = 0.57.

## Discussion

The purpose of this study was to investigate whether a subset of ASD with expressive language impairment could modify their motor control strategy during a simple activity, two-legged hopping in place, in the presence of an auditory cue. It was hypothesized that H(1) the ELI-ASD group would be able to successfully complete a two-legged hopping in place task at a self-selected cadence, but H(2) the ELI-ASD group would not be able to match their hopping cadence to an external auditory cue while all control participants would be within 5% of the cued frequency and that H(3) there would be a range of responses within the ELI-ASD population.

### Self-selected hopping cadence

The first hypothesis was accepted as both groups were able to successfully complete two 15-s two legged hopping trials on a force plate at a self-selected cadence. When comparing the first to second trial cadences, the ELI-ASD group demonstrated similar variances as the control group. This indicated that the movement pattern was as stable as an age-matched control. Furthermore, the groups were not significantly different from one another and computed cadences were similar to those reported by Farley et al. (1991), 2.21 ± 0.07 hops·s^−1^, but larger than those reported by Rousanoglou and Boudolos (2006). Many common diagnostic movement batteries (e.g., Bruininks-Oseretsky Test of Motor Proficiency, Movement Battery for Children 2) used to diagnose motor function include variants of two-legged hopping within their testing battery (Henderson et al., 1992; Bruininks and Bruininks, 2005). Two-legged hopping is also found in many elementary physical education models as it teaches gross multi-joint coordination by recruiting large hip, knee and ankle extensor musculature that leads to developmental progression in many dynamic game skills (Gallahue and Donnelly, 2003). Although a fundamental movement skill, it requires motor coordination, dynamic balance, and core stability. Considering the current study only assessed severely autistic individuals, two-legged hopping in place would appear to be a feasible movement screen for most individuals diagnosed in the spectrum.

### Effect of external auditory cue

The second hypothesis was accepted as two-legged hopping in place in a sample of ELI-ASD individuals was significantly altered at the 3.0 Hz condition from an age-matched control group that was able to match cadence when an auditory cue was provided. Participants in the ELI-ASD group were not able to significantly alter their hopping cadence in the presence of an auditory cue and were unsuccessful in modifying motor output 67% of the time. Conversely, all participants in the control group were able to match either a 2.3 or 3.0 Hz auditory cue. When two-legged hopping is matched to an external auditory cue, the task becomes multi-modal. Auditory processing must be integrated with proper motor cortex commands that are refined via proprioceptive sensory feedback from muscle spindles and golgi tendon organs in order to match hopping cadence to this external cue. O'Neill and Jones (1997) report accounts of autistic individuals have difficulty processing simultaneous sensory modes. When sensory information converges from multiple sources, it must be integrated or weighted in such a way that the uncertainty of the resulting neural output is minimized (van Beers et al., 2002).

The results of the current study confirm contemporary views of potential sensory processing deficiencies in an ELI-ASD population. The inability of the ELI-ASD group to match their hop timing to an auditory cue can be attributed to a deficiency with processes sensing auditory cues. Although reports on auditory brainstem response have been varied, there appears to be evidence suggesting impaired early auditory pathways (Marco et al., 2011). Some studies have reported longer latencies in individuals with ASD which may indicate slower neural conduction velocities. Although the mechanism of ASD auditory processing deficiency is still not clear and not in the scope of the current study, it is possible that a delayed auditory processing may have influenced the timing of motor neuron transmission in our participants with ELI-ASD.

The current study's task, two-legged hopping in synch with an auditory cue, also requires appropriately timed motor and proprioceptive responses. When compared to intellect, language abilities and emphatic abilities, autistic individuals are most impaired in their motor coordination, specifically gross motor coordination (Piek and Dyck, 2004). Therefore, the results of the current study which investigated a gross motor skill, two-legged hopping, in a group of individuals diagnosed with autism and limited language abilities are not surprising. One possible explanation is that participants with ELI-ASD were unable to alter the stiffness of musculotendons crossing the ankle, knee, and hip. In order to hop at greater frequencies it requires an increase in effective leg stiffness. Leg stiffness can be modulated by altering musculotendon tensions which in turn alter joint stiffness (Johns and Wright, 1962; Riemann and Lephart, 2002). Increased gamma motor neuronal activity, from either sensory input or supraspinal drive, alters muscle spindle sensitivity and ultimately refines musculotendon tension. Individuals with autism have been previously shown to rely on proprioceptive feedback (distal) more than visual/auditory (proximal) sources of information (Masterton and Biederman, 1983), and proprioception during a mono-articulate reaching task was not impaired as compared to children who are typically developing (Fuentes et al., 2011). These findings, in the context of this study, could suggest that the increased weighting of proprioceptive information (distal) in creating an internal motor model of hopping is challenged to integrate simultaneous auditory cues (proximal) to refine motor control strategy.

Alternative explanations to the lack of hopping success in participants with ELI-ASD could be attributed either to (1) cognitive demands and/or (2) task complexity. It could be argued that the ELI-ASD did not comprehend the task and thus cognitive inabilities rather than any sensory processing deficiency explained their performance. However, the ELI-ASD did have an average increase in hopping cadence and effective leg stiffness from the 2.3 Hz trials as compared to the 3.0 Hz trials, although these increases was not deemed significant. This would suggest that there may have been an attempt to modify hopping strategy to meet the external cue, but a limited subject pool (*n* = 6) may have prevented this effect from reaching significance. Hopping in synch with at a cadence of 3 hops·s^−1^ requires motor precision and substantial muscular strength across the ankle, knee, and hip. Kern et al. (2011) recently demonstrated that CARS level was a significant predictor of max hand grip strength. CARS level and hand grip strength were negatively related, so as CARS level rise max hand grip strength decreases. Lower extremity muscular strength was not assessed in our study so it is possible that performance could be attributed to an inability to produce muscular demands necessary to match the auditory cue.

### Movement criterion

The third hypothesis was accepted as the six participants with ELI-ASD demonstrated a range of hopping cadencies when attempting to match auditory cues, while control subjects were nearly perfect. One ELI-ASD was successful in 3 of the 4 cued trials while the remaining five participants experienced varied deviation (5.6–42.2%). Although a relatively easy task for control subjects, this multi-modal task provided a heterogeneous response in a limited ELI-ASD population.

The majority of reports on ASD motor qualities use a wide breadth of participants across the spectrum. Exceptions to this would be studies that are inclusive to either Asperger's syndrome or high-functioning individuals. Typically these classifications are based on self-reports or observational analyses by trained professionals. Despite efforts made to discretize the population by these analyses, it is likely that heterogeneity would remain in regards to sensory processing deficits (Ben-Sasson et al., 2008). Furthermore, several have recommended the need for improved classification in an effort to elucidate neurological underpinnings with specific characteristics (Verhoeven et al., 2010; Marco et al., 2011). Quantitative movement screens, potentially like the two-legged hopping task in the current study, may provide an improved classification system for inclusion criteria in studies. At minimum, quantitative movement screens can more definitively be utilized to evaluate functional movement outcomes pre- and post-interventions (Bhat et al., 2011).

Several limitations should be noted regarding this experiment when considering the applicability of results to other populations. ELI-ASD group size was limited based on stringent inclusion criteria and a 33% experimental mortality rate. These limitations resulted in respective effect sizes for the 2.3 and 3.0 Hz trials of 0.31 and 0.75. This indicated only small group differences for the 2.3 Hz trials and moderate differences for the 3.0 Hz trials. Because of the multi-modal nature of the task, it is difficult to prescribe which sensory mode may be deficient. As has been previously noted, these findings cannot be applied to other subsets in ASD as sensory processing deficits appear heterogeneously across the spectrum. Since our participants had limited language abilities, it was difficult to confirm complete comprehension of the task. Researchers assumed they understood our verbal commands and visual modeling of the activity, but we have not assurances of this.

## Conclusions

Sensory processing deficits are common in ASD and multi-modal tasks present unique challenges to this population. The current investigation confirmed an impaired motor control strategy during an auditory-cued two-legged hopping task in an under-studied subset of ASD, individuals with expressive language impairments. An age-matched control group was nearly perfect in their performance, however, the ELI-ASD group had a varied deviant response indicating the possibly utility of this task for an improved movement classification tool. The findings would suggest that an ELI-ASD population on the cusp of adulthood present with an impaired motor control strategy that will influence adult movement patterns and perhaps warrant continued therapies. As this work was constrained to a small ASD subset at the end of the pediatric scale, future investigations should both extend to include participants across the spectrum and at younger age intervals. Such work would elicit the relationship of motor response to both autistic level and age. Modifications of the current multimodal task should extend to the use of a visual cue in addition to an auditory one and extend the cued frequency to a larger range. Additionally, the inclusion of enhanced metrics, such as the variability of within trial inter-hop intervals, may provide further evidence of ASD neurological underpinnings. As movement aberrations in the autistic population become more accepted as a core feature as opposed to a co-morbidity, movement screens may offer an improved opportunity to identify sensory processing deficiencies to both improve neurological underpinnings and intervention therapies.

### Conflict of interest statement

The authors declare that the research was conducted in the absence of any commercial or financial relationships that could be construed as a potential conflict of interest.
